# Supplementary figures and images for: Inhibition of the nucleolar RNA exosome facilitates adaptation to starvation
Source: PLoS Biol. 2025 May 21;23(5):e3003190. doi: 10.1371/journal.pbio.3003190 (PMC12136472; doi:10.1371/journal.pbio.3003190)

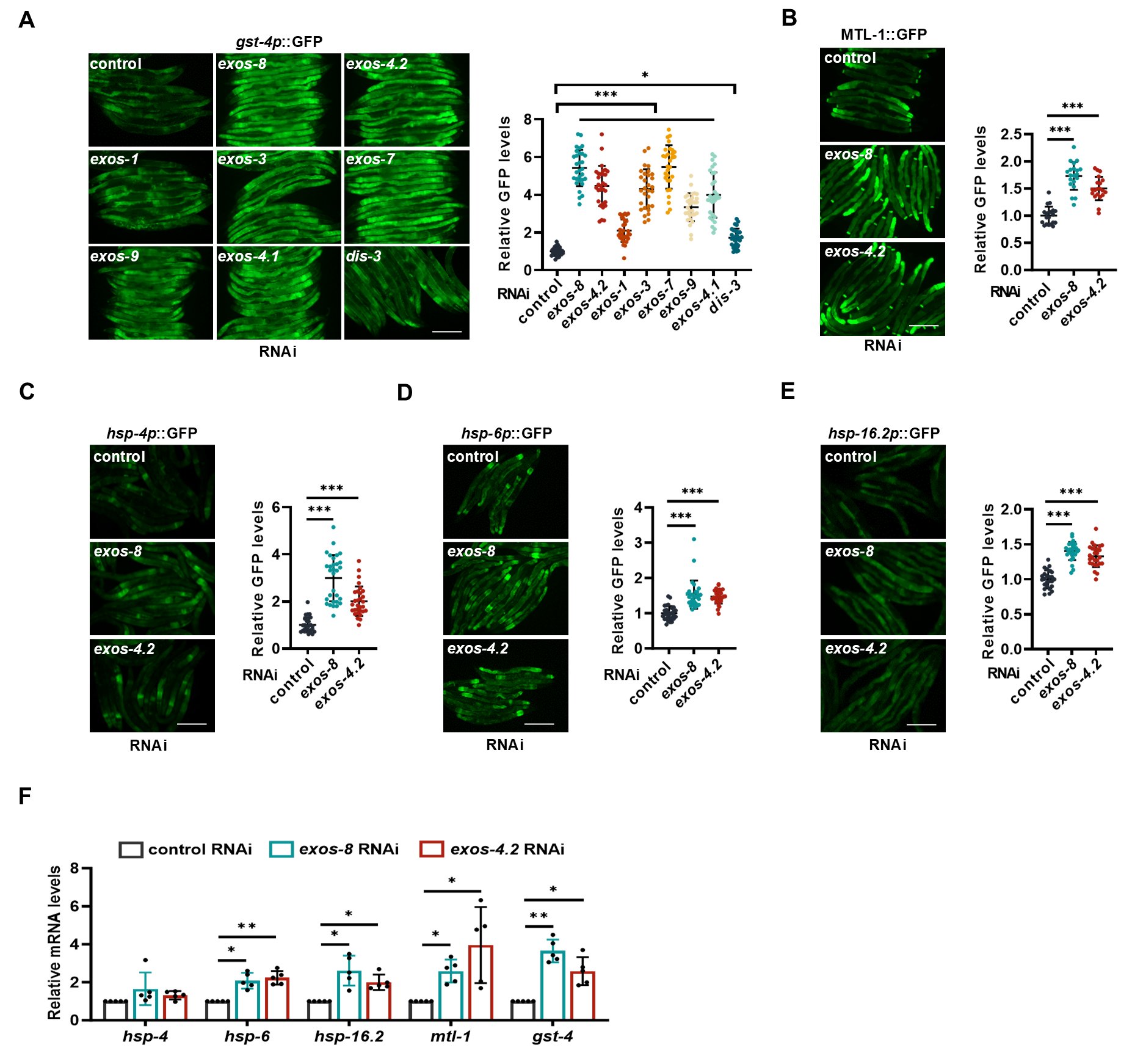

Supplement: S1 Fig — (A) Left: RNAi effects of the RNA exosome subunits on gst-4p::GFP expression. Scale bar = 200 μm. Right: Relative GFP intensity. One-way ANOVA with Dunnett’s multiple comparisons test (*p = 0.0129) (n = 30 worms). (B–E) Left: Effects of exos-8 and exos-4.2 RNAi on the expression of MTL-1::GFP (B), hsp-4p::GFP (C), hsp-6p::GFP (D), and hsp-16.2p::GFP (E). These reporter constructs express GFP under the control of endogenous promoters from individual stress-response genes. Scale bar = 200 μm. Right: Relative GFP intensity. One-way ANOVA with Dunnett’s multiple comparisons test (n = 20−30 worms). (F) Effects of exos-8 and exos-4.2 RNAi on the mRNA levels of stress response genes. Multiple t test with correction for multiple comparisons using the Holm–Sidak method (**p = 0.0076 for hsp-6, 0.003 for gst-4, *p = 0.0168 for hsp-6, 0.0202/0.021 for hsp-16.2, 0.0168/0.0589 for mtl-1,0.0255 for gst-4) (n = 5 independent experiments). The numerical data presented in this figure can be found in S1 Data. Data are presented as mean ± SD. *p < 0.05, **p < 0.01, ***p < 0.001. (TIF) [file pbio.3003190.s001.tif]

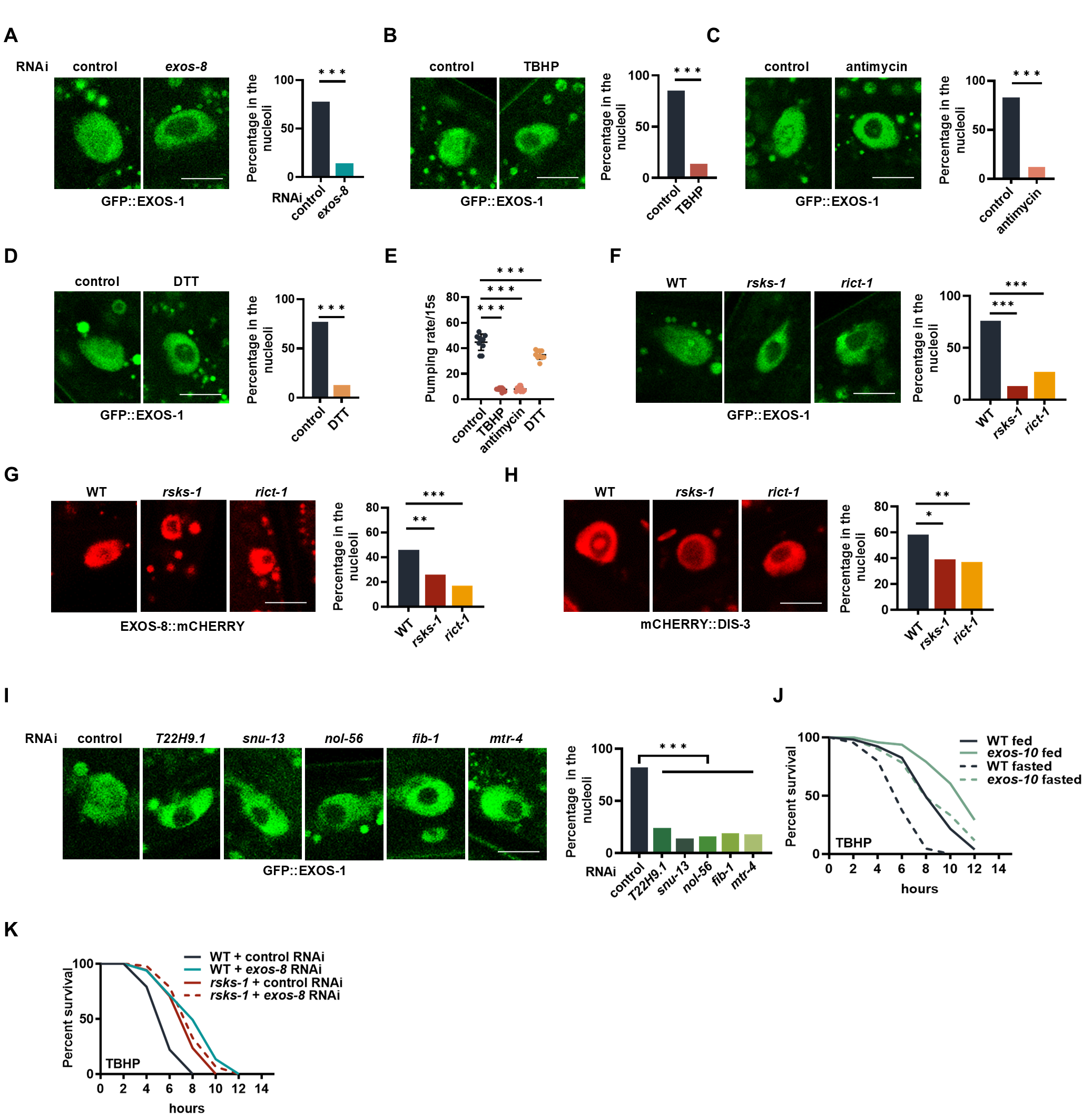

Supplement: S2 Fig — (A) Effects of exos-8 RNAi on the nucleolar localization of GFP::EXOS-1 in intestinal cells. Scale bar = 10 μm. Right: Percentage of fluorescent signals in the nucleoli. n = 78 and 93 cells. (B–D) Effects of oxidative stress (B), mitochondrial stress (C), and endoplasmic reticulum stress (D) on the nucleolar localization of GFP::EXOS-1 in intestinal cells. Scale bar = 10 μm. Right: Percentage of fluorescent signals in the nucleoli. n = 75−127 cells. (E) Effects of stresses on food pumping rate. One-way ANOVA (n = 10 worms). (F–H) Left: Effects of the rsks-1 and rict-1 mutations on the nucleolar localization of GFP::EXOS-1 (F), EXOS-8:: mCHERRY (**p = 0.005) (G), and mCHERRY::DIS-3 (*p = 0.0107, **p = 0.0045) (H) in intestinal cells. Scale bar = 10 μm. Right: Percentage of fluorescent signals in the nucleoli. n = 78−171 cells. (I) Left: The nucleolar localization of GFP::EXOS-1 in intestinal cells in response to RNAi targeting T22H9.1, snu-13, nol-56, fib-1, and mtr-4. Scale bar = 10 μm. Right: Percentage of fluorescent signals in the nucleoli. n = 93−109 cells. (J) Effect of the exos-10 mutation on oxidative stress resistance in well-fed and 12-h starved L4-stage nematodes. (K) Effects of the rsks-1 mutation on exos-8 RNAi-induced oxidative stress resistance. Data are presented as mean ± SD. *p < 0.05, **p < 0.01, ***p < 0.001. S1 Table provides all repeats and statistical analyses of the survival experiments, where Repeat 1 of each experiment was used for generating the graphs. The numerical data presented in this figure can be found in S1 Data. (TIF) [file pbio.3003190.s002.tif]

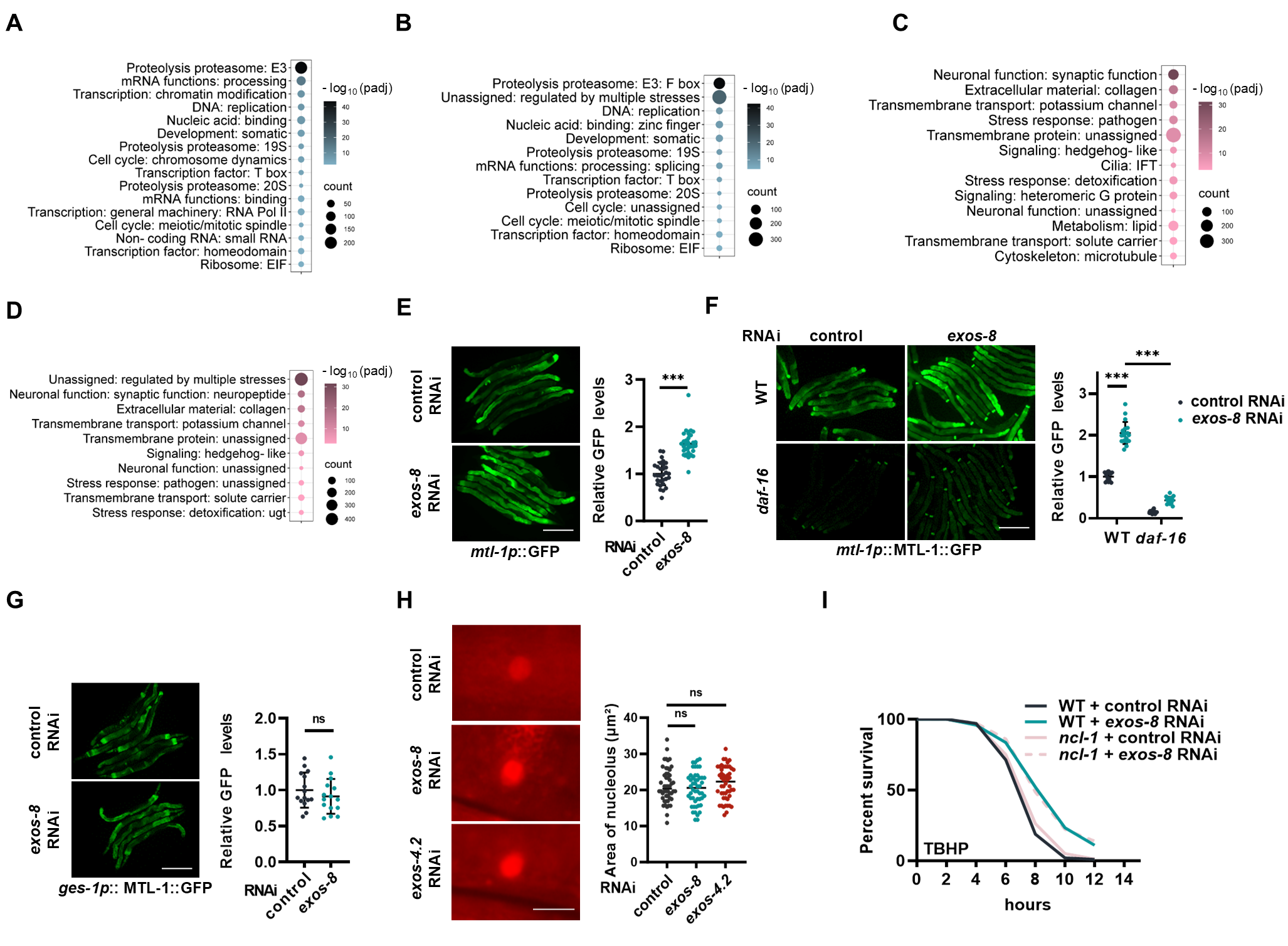

Supplement: S3 Fig — (A, B) Functional classification of exos-8 RNAi-downregulated genes by WormCat Categories Two (A) and Three (B) analysis. (C, D) Functional classification of exos-8 RNAi-upregulated genes by WormCat Categories Two (C) and Three (D) analysis. (E–G) Left: Effects of exos-8 RNAi on the mtl-1 transcriptional reporter (mtl-1p::GFP) (E), translational reporter (mtl-1p::MTL-1::GFP) (F), and ectopic expression reporter (ges-1p::MTL-1::GFP) (G). The daf-16 mutation suppresses the induction of the translational reporter in exos-8(RNAi) worms (F). Scale bar = 200 µm. Right: Relative GFP intensity. E and G, unpaired two-tailed t test. F, two-way ANOVA with Turkey’s multiple comparisons test (n = 14−30 worms). (H) Left: Effects of exos-8 and exos-4.2 RNAi on nucleolus size indicated by FIB-1::mCHERRY in intestinal cells. Scale bar = 10 μm. Right: Quantification of nucleolus size. One-way ANOVA with Dunnett’s multiple comparisons test (n = 50 cells). (I) Effect of ncl-1 mutation on exos-8 RNAi-induced oxidative stress resistance. Data are presented as mean ± SD. ***p < 0.001. S1 Table provides all repeats and statistical analyses of the survival experiments, where Repeat 1 of each experiment was used for generating the graphs. The numerical data presented in this figure can be found in S1 Data. (TIF) [file pbio.3003190.s003.tif]

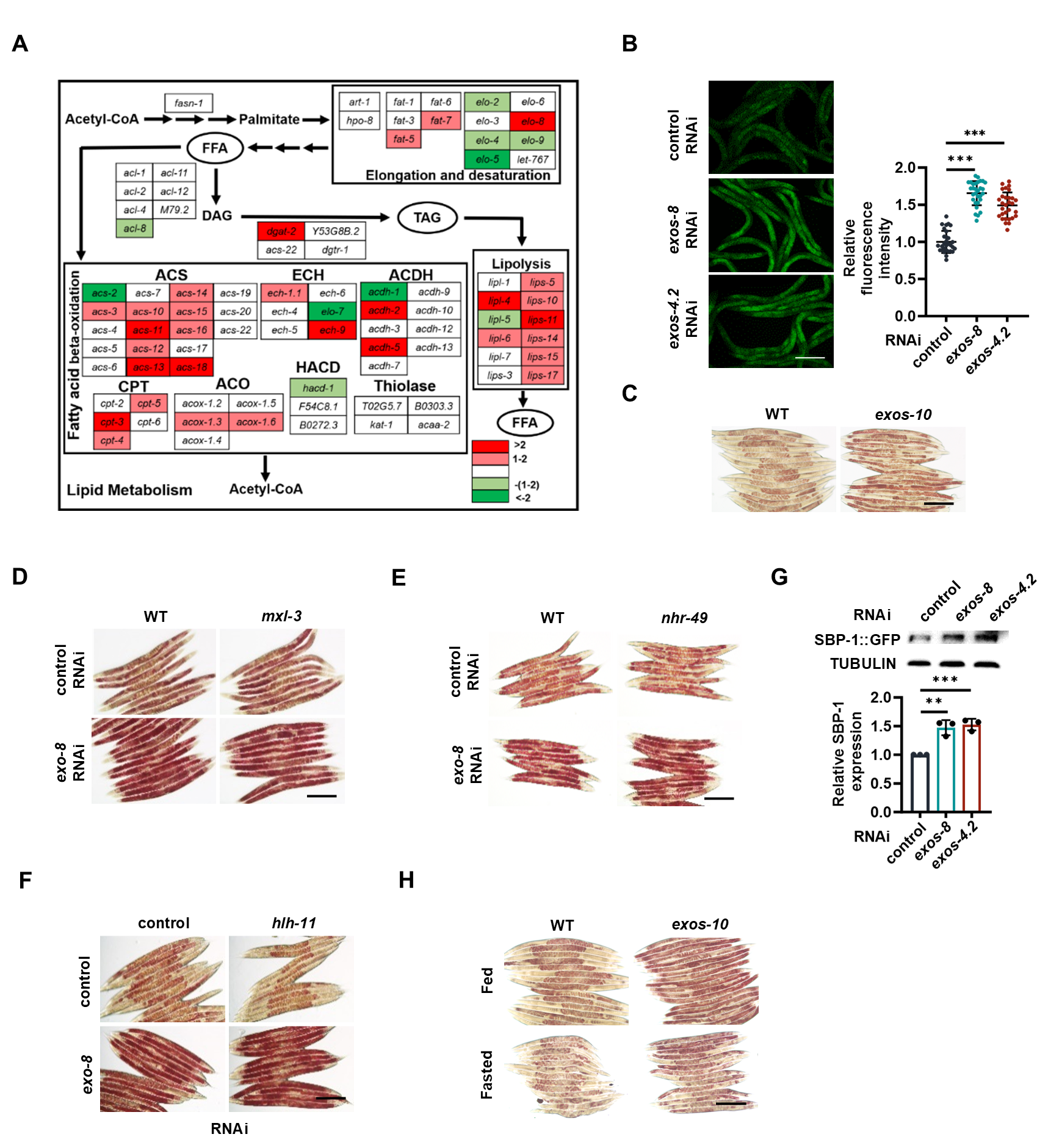

Supplement: S4 Fig — (A) Effects of exos-8 RNAi on the expression of lipid metabolic genes based on RNA-seq data. Fold changes were shown by colors as indicated in fig. FFA: free fatty acid; DAG: diglycerides; TAG: triglycerides; ACS: acyl-CoA synthase; ECH: enoyl CoA hydratase; ACDH: acyl-CoA dehydrogenase; CPT: carnitine palmityl transferase; ACO: acyl-CoA oxidase; HACD: hydroxy acyl-CoA dehydrogenase. (B) Left: Effects of exos-8 and exos-4.2 RNAi on fat accumulation measured by Nile red staining. Scale bar = 200 µm. Right: Relative Nile red intensities. One-way ANOVA with Dunnett’s multiple comparisons test (n = 30 worms). (C) Left: Effect of the exos-10 mutation on fat accumulation measured by Oil red O staining. Scale bar = 200 μm. (D–F) Effects of the mxl-3 mutation (D), the nhr-49 mutation (E), and hlh-11 RNAi (F) on exos-8 RNAi-induced lipid accumulation. Scale bar = 200 µm. (G) Upper: Effects of exos-8 and exos-4.2 RNAi on the protein levels of SBP-1::GFP. Lower: Quantification of western blot. One-way ANOVA with Dunnett’s multiple comparisons test (**p = 0.0017) (n = 3 experiments). (H) Effect of the exos-10 mutation on lipid accumulation in well-fed and starved animals. Data are presented as mean ± SD. *p < 0.05, ***p < 0.001. The numerical data presented in this figure can be found in S1 Data. (TIF) [file pbio.3003190.s004.tif]

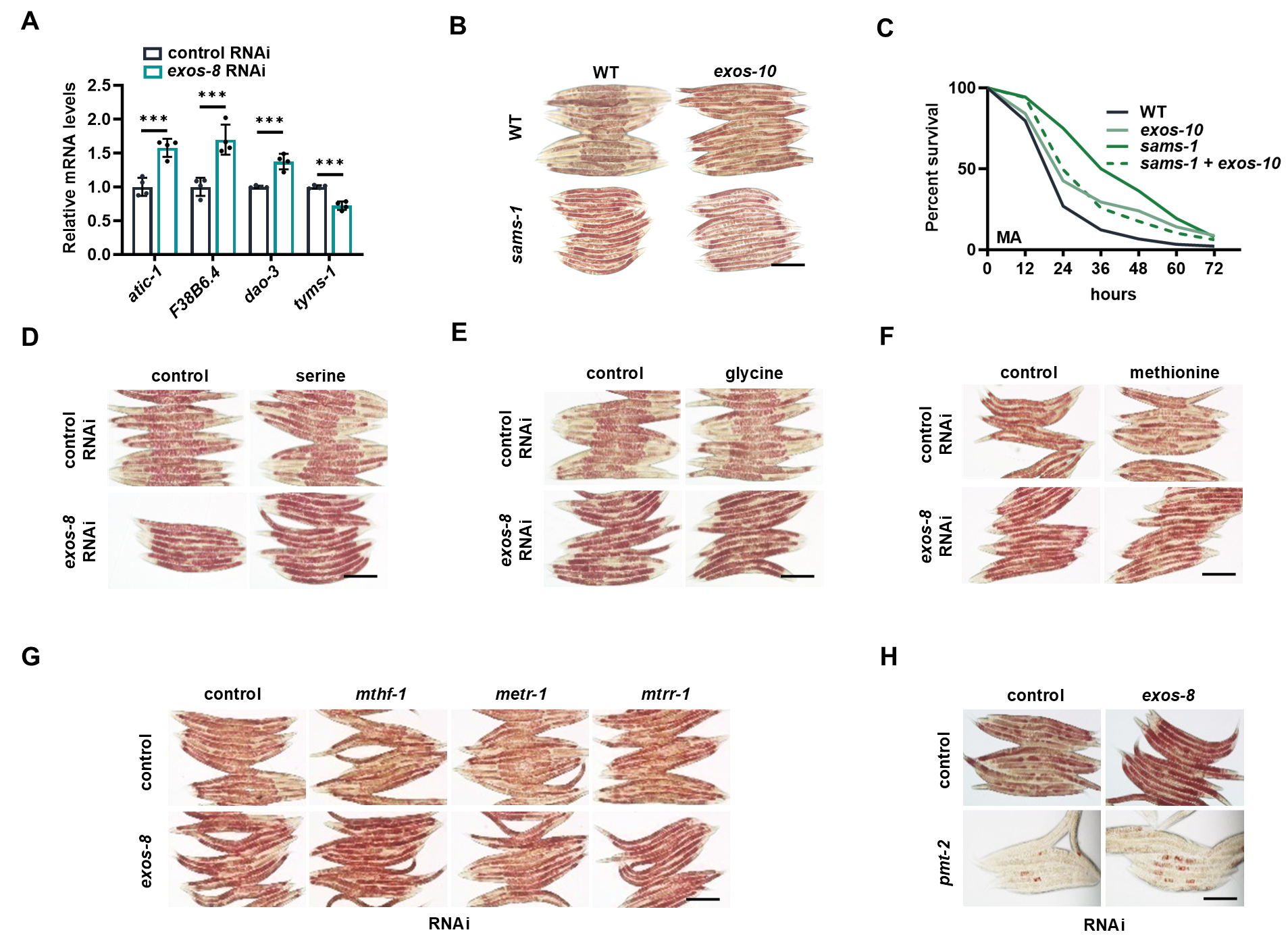

Supplement: S5 Fig — (A) RNA-seq data reveal regulation of nucleotide metabolic enzymes by exos-8 RNAi. False Discovery Rate (FDR) calculated by the Benjamini and Hochberg’s approach (n = 4 experimental groups). (B) Effect of the sams-1 mutation on exos-10 mutation-induced lipid accumulation. Scale bar = 200 μm. (C) Effect of the sams-1 mutation on exos-10 mutation-induced mitochondrial stress resistance. (D–F) Supplementations of serine (D), glycine (E), or methionine (F) have no effects on exos-8 RNAi-induced lipid accumulation. Scale bar = 200 μm. (G) Knockdowns of the 1CM genes have little or no effects on exos-8 RNAi-induced lipid accumulation. Scale bar = 200 μm. (H) Knockdown of pmt-2 inhibits exos-8 RNAi-induced lipid accumulation. Scale bar = 200 μm. Data are presented as mean ± SD. **p < 0.01, ***p < 0.001. S1 Table provides all repeats and statistical analyses of the survival experiments, where Repeat 1 of each experiment was used for generating the graphs. The numerical data presented in this figure can be found in S1 Data. (TIF) [file pbio.3003190.s005.tif]

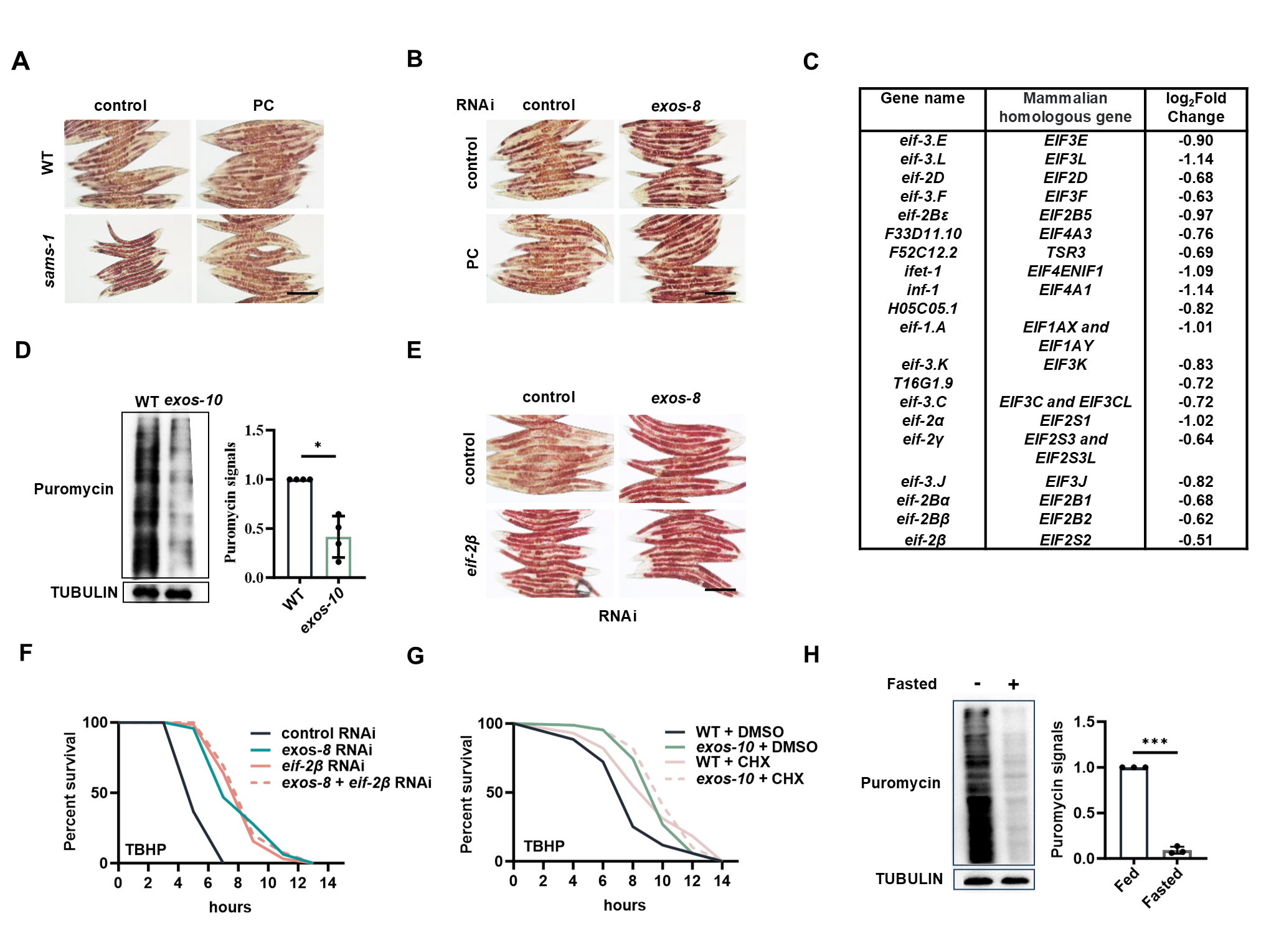

Supplement: S6 Fig — (A) Effects of PC supplementation on lipid accumulation in sams-1 mutants. Scale bar = 200 μm. (B) Effects of PC supplementation on exos-8 RNAi-induced lipid accumulation. Scale bar = 200 µm. (C) RNA-seq data reveal translation initiation factors that were downregulated by exos-8 RNAi. (D) Left: Effect of the exos-10 mutation on translation. Right: Quantification of translation. Paired two-tailed t test (*p = 0.0116) (n = 4 experiments). (E, F) Effects of eif-2β RNAi on exos-8 RNAi-induced lipid accumulation (D) and oxidative stress resistance (E). Scale bar = 200 μm. (G) Effect of the translation inhibitor cycloheximide (CHX) on exos-10 mutation-induced oxidative stress resistance. (H) Left: Starvation suppresses cellular translation. Right: Quantification of translation. t test (n = 3 experiments). Data are presented as mean ± SD. * p < 0.05, ***p < 0.001. S1 Table provides all repeats and statistical analyses of the survival experiments, where Repeat 1 of each experiment was used for generating the graphs. The numerical data presented in this figure can be found in S1 Data. (TIF) [file pbio.3003190.s006.tif]

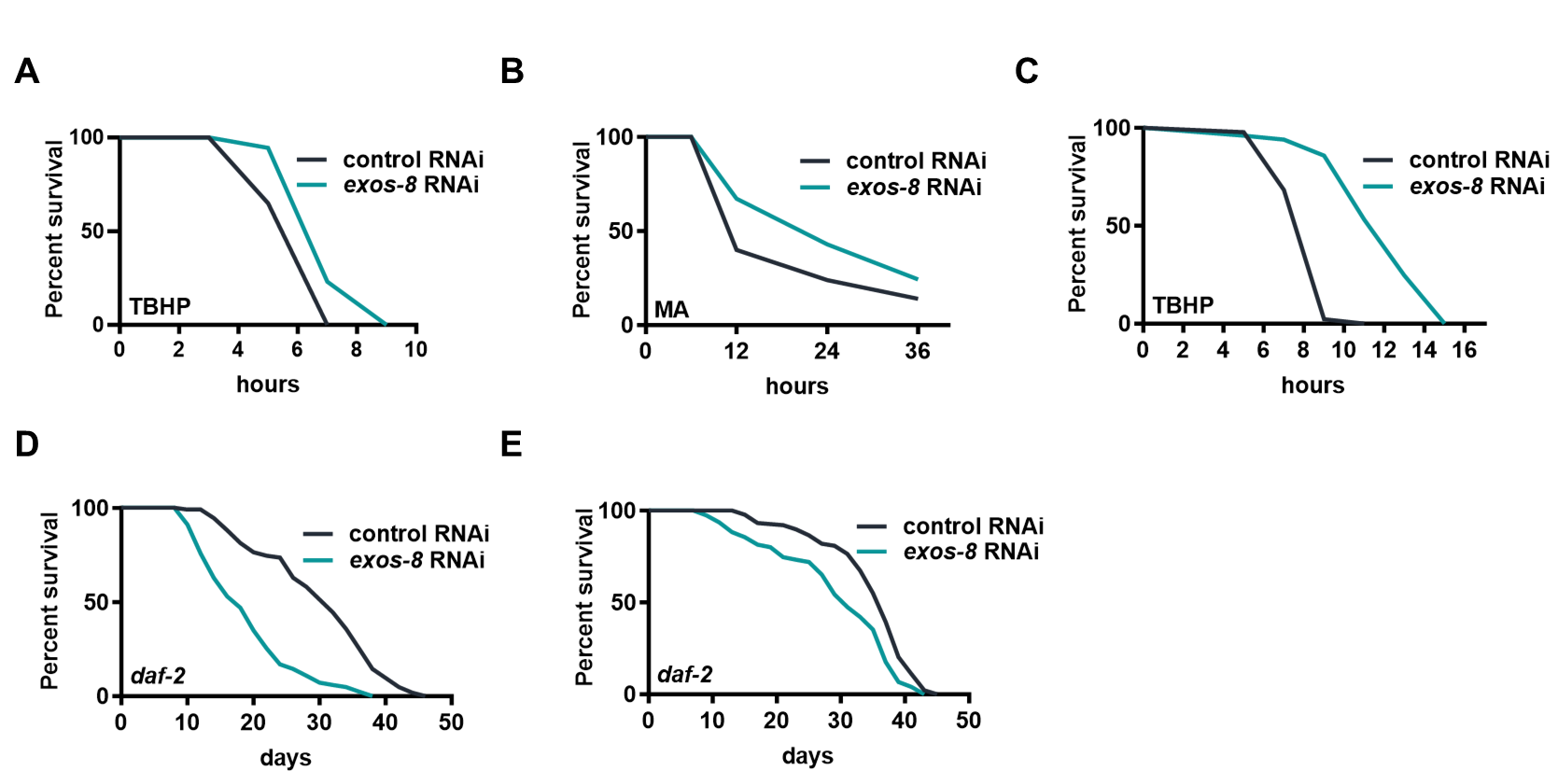

Supplement: S7 Fig — (A, B) Effects of post-developmental exos-8 RNAi on oxidative stress resistance (A) and mitochondrial stress resistance (B). (C) Effects of exos-8 RNAi on oxidative stress resistance in daf-2 mutants. (D, E) Effects of exos-8 RNAi from birth (D) and after development (E) on the life span of daf-2 mutants. S1 and S2 Tables provide all repeats and statistical analyses of the survival experiments, where Repeat 1 of each experiment was used for generating the graphs. (TIF) [file pbio.3003190.s007.tif]

**Figure S4G**

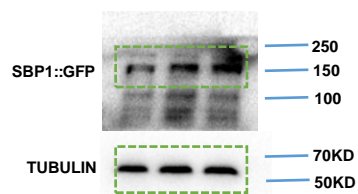

**Figure 5A**

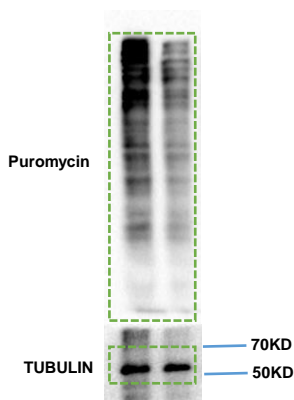

**Figure 5B**

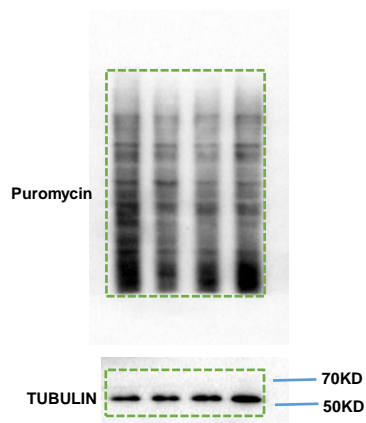

**Figure 5C**

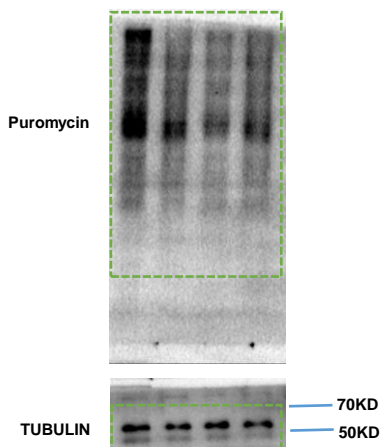

**Figure 5D**

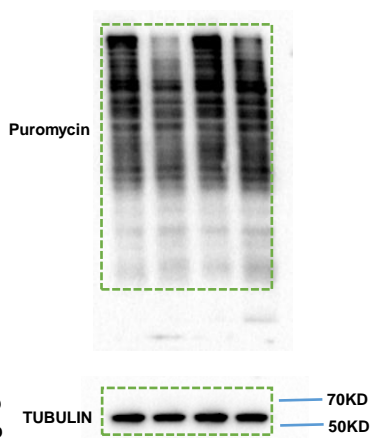

**Figure 6F**

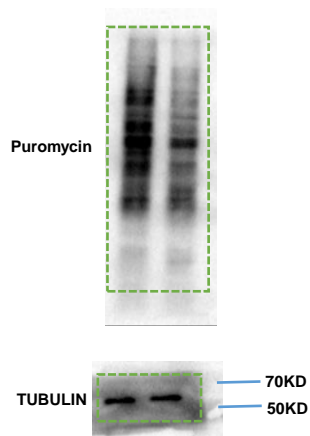

**Figure S6D**

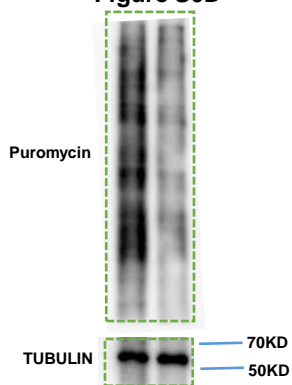

**Figure S6H**

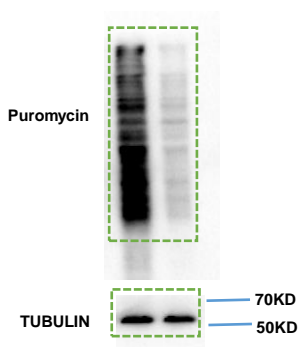

Figure 2F

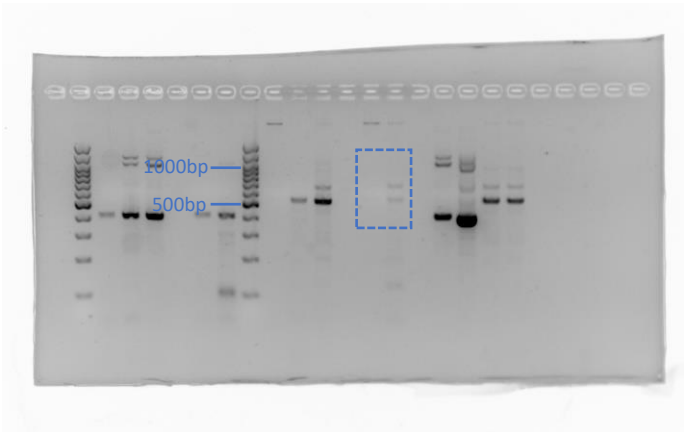

Figure 2G

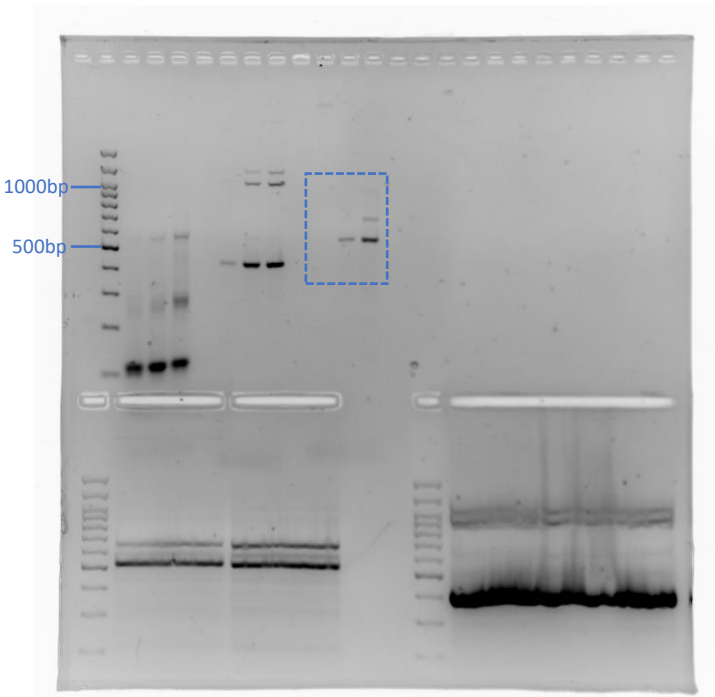

Supplement: S1 Raw Images — (PDF) [file pbio.3003190.s014.pdf]
