# Supplementary material for: Inhibition of the nucleolar RNA exosome facilitates adaptation to starvation
Source: PLoS Biol. 2025 May 21;23(5):e3003190. doi: 10.1371/journal.pbio.3003190 (PMC12136472; doi:10.1371/journal.pbio.3003190)
Supplement: S1 Table — Results shown are representative of at least two independent experiments. Repeats 1 were graphed in figures. (DOCX) [file pbio.3003190.s008.docx]

**S1 Table. Survival data. Results shown are representative of at least two independent experiments. Repeats 1 were graphed in Figures.**

| Figures | Strain/Treatment | Mean Lifespan  ± SEM (hours) | # Worms | p value |
| --- | --- | --- | --- | --- |
| **1C** |  |  |  |  |
| Repeat 1 | Control RNAi | 6.89 ± 0.12 | 50 |  |
|  | *exos-8* RNAi | 9.57 ± 0.36 | 51 | **<0.001^a^** |
|  | *exos-4.2* RNAi | 10.02 ± 0.32 | 57 | **<0.001^a^** |
| Repeat 2 | Control RNAi | 7.75 ± 0.2 | 43 |  |
|  | *exos-8* RNAi | 12.62 ± 0.29 | 56 | **<0.001^a^** |
|  | *exos-4.2* RNAi | 12.21 ± 0.38 | 51 | **<0.001^a^** |
| Repeat 3 | Control RNAi | 7.62 ± 0.21 | 40 |  |
|  | *exos-8* RNAi | 11.83 ± 0.57 | 41 | **<0.001^a^** |
|  | *exos-4.2* RNAi | 10.16 ± 0.52 | 41 | **<0.001^a^** |
| **1D** |  |  |  |  |
| Repeat 1 | Control RNAi | 4.32 ± 0.67 | 58 |  |
|  | *exos-8* RNAi | 11.23 ± 1.32 | 55 | **<0.001^a^** |
|  | *exos-4.2* RNAi | 12.27 ± 1.31 | 57 | **<0.001^a^** |
| Repeat 2 | Control RNAi | 3.99 ± 0.62 | 54 |  |
|  | *exos-8* RNAi | 9.43 ± 1.03 | 50 | **<0.001^a^** |
|  | *exos-4.2* RNAi | 11.94 ± 1.33 | 51 | **<0.001^a^** |
| Repeat 3 | Control RNAi | 3.56 ± 0.39 | 51 |  |
|  | *exos-8* RNAi | 12.89 ± 1.28 | 54 | **<0.001^a^** |
|  | *exos-4.2* RNAi | 12.45 ± 1.36 | 53 | **<0.001^a^** |
| **1E** |  |  |  |  |
| Repeat 1 | Control RNAi | 13.75 ± 0.41 | 90 |  |
|  | *exos-8* RNAi | 26.61 ± 1.46 | 72 | **<0.001^a^** |
|  | *exos-4.2* RNAi | 25.55 ± 1.9 | 49 | **<0.001^a^** |
| Repeat 2 | Control RNAi | 19.4 ± 1.05 | 53 |  |
|  | *exos-8* RNAi | 35.96 ± 1.89 | 57 | **<0.001^a^** |
|  | *exos-4.2* RNAi | 36.3 ± 2.76 | 58 | **<0.001^a^** |
| Repeat 3 | Control RNAi | 11.51± 0.69 | 48 |  |
|  | *exos-8* RNAi | 22.13 ± 1.41 | 49 | **<0.001^a^** |
|  | *exos-4.2* RNAi | 20.11 ± 1.6 | 51 | **<0.001^a^** |
| **1F** |  |  |  |  |
| Repeat 1 | Control RNAi | 10.52 ± 0.24 | 72 |  |
|  | *exos-8* RNAi | 13.72 ± 0.36 | 58 | **<0.001^a^** |
|  | *exos-4.2* RNAi | 11.97 ± 0.28 | 73 | **<0.001^a^** |
| Repeat 2 | Control RNAi | 6.33 ± 0.13 | 51 |  |
|  | *exos-8* RNAi | 10.99 ± 0.35 | 53 | **<0.001^a^** |
|  | *exos-4.2* RNAi | 9.65 ± 0.37 | 44 | **<0.001^a^** |
| Repeat 3 | Control RNAi | 9.42 ± 0.12 | 52 |  |
|  | *exos-8* RNAi | 11.21 ± 0.3 | 46 | **<0.001^a^** |
|  | *exos-4.2* RNAi | 11.53 ± 0.25 | 61 | **<0.001^a^** |
| 1G |  |  |  |  |
| Repeat 1 | WT | 6.13 ± 0.21 | 70 |  |
|  | ***exos-10*** | **8.94 ± 0.38** | **48** | **<0.001^b^** |
| Repeat 2 | WT | 7.68 ± 0.29 | 110 |  |
|  | *exos-10* | 9.15 ± 0.28 | 90 | **<0.001^b^** |
| 1H |  |  |  |  |
| Repeat 1 | **WT** | **26.17 ± 1.75** | **98** |  |
|  | ***exos-10*** | **41.99 ± 26.9** | **104** | **<0.001^b^** |
| Repeat 2 | **WT** | **26.59 ± 2.27** | **93** |  |
|  | ***exos-10*** | **36.97 ± 2.55** | **97** | **0.0013^b^** |
| 1I |  |  |  |  |
| Repeat 1 | **WT** | **5.75 ± 0.4** | **94** |  |
|  | ***exos-10*** | **9.68 ± 0.25** | **147** | **<0.001^b^** |
| Repeat 2 | **WT** | **6.23 ± 0.46** | **70** |  |
|  | ***exos-10*** | **9.45 ± 0.47** | **92** | **<0.001^b^** |
| 1J |  |  |  |  |
| Repeat 1 | **WT** | **5.52 ± 0.21** | **118** |  |
|  | ***exos-10*** | **9.8 ± 0.22** | **139** | **<0.001^b^** |
| Repeat 2 | **WT** | **7.65 ± 0.3** | **88** |  |
|  | ***exos-10*** | **10.85 ± 0.24** | **71** | **<0.001^b^** |
| 2H |  |  |  |  |
| Repeat 1 | Control RNAi | 9.12 ± 0.31 | 42 |  |
|  | *T22H9.1* RNAi | 12.23 ± 0.29 | 46 | **<0.001^a^** |
|  | *nol-56* RNAi | 11.74 ± 0.4 | 47 | **<0.001^a^** |
|  | *fib-1* RNAi | 12.77 ± 0.41 | 49 | **<0.001^a^** |
|  | *mtr-4* RNAi | 13.12 ± 0.36 | 48 | **<0.001^a^** |
| Repeat 2 | Control RNAi | 7.11 ± 0.19 | 49 |  |
|  | *T22H9.1* RNAi | 10.75 ± 0.38 | 39 | **<0.001^a^** |
|  | *nol-56* RNAi | 10.02 ± 0.34 | 43 | **<0.001^a^** |
|  | *fib-1* RNAi | 9.68 ± 0.26 | 51 | **<0.001^a^** |
|  | *mtr-4* RNAi | 9.22 ± 0.25 | 53 | **<0.001^a^** |
| Repeat 3 | Control RNAi | 8.35 ± 0.27 | 48 |  |
|  | *T22H9.1* RNAi | 11.88 ± 0.36 | 41 | **<0.001^a^** |
|  | *nol-56* RNAi | 9.4 ± 0.27 | 51 | **0.0469^a^** |
|  | *fib-1* RNAi | 9.98 ± 0.28 | 58 | **<0.001^a^** |
|  | *mtr-4* RNAi | 10.66 ± 0.31 | 44 | **<0.001^a^** |
| 2I |  |  |  |  |
| Repeat 1 | Fed control RNAi | 7.74 ± 0.40 | 53 |  |
|  | Fed *exos-8* RNAi | 12.41 ± 0.51 | 52 | **<0.001^a^** |
|  | Fasted control RNAi | 4.34 ± 0.22 | 49 | **<0.001^b^** |
|  | Fasted *exos-8* RNAi | 11.84 ± 0.48 | 62 | <0.001^c^ |
| Repeat 2 | Fed control RNAi | 7.95 ± 0.41 | 48 |  |
|  | Fed *exos-8* RNAi | 12.75 ± 0.49 | 46 | **<0.001^a^** |
|  | Fasted control RNAi | 4.18 ± 0.21 | 48 | **<0.001^b^** |
|  | Fasted *exos-8* RNAi | 12.43 ± 0.45 | 55 | **<0.001^c^** |
| Repeat 3 | Fed control RNAi | 6.38 ± 0.41 | 40 |  |
|  | Fed *exos-8* RNAi | 12.6 ± 0.57 | 43 | **<0.001^a^** |
|  | Fasted control RNAi | 4.18 ± 0.19 | 58 | **<0.001^b^** |
|  | Fasted *exos-8* RNAi | 11.42 ± 0.54 | 54 | **<0.001^c^** |
| S2J |  |  |  |  |
| Repeat 1 | **WT Fed** | **8.87 ± 0.34** | **55** |  |
|  | ***exos-10* Fed** | **10.58 ± 0.31** | **50** | **<0.001^b^** |
|  | **WT Fasted** | **6.35 ± 0.23** | **66** | **<0.001^a^** |
|  | ***exos-10* Fasted** | **8.97 ± 0.35** | **63** | **<0.001^c^** |
| Repeat 2 | **WT Fed** | **9.12 ± 0.29** | **79** |  |
|  | ***exos-10* Fed** | **10.51 ± 0.23** | **73** | **<0.001^b^** |
|  | **WT Fasted** | **6.24 ± 0.2** | **74** | **<0.001^a^** |
|  | ***exos-10* Fasted** | **9.1 ± 0.42** | **54** | **<0.001^c^** |
| S2K |  |  |  |  |
| Repeat 1 | WT + control RNAi | 6.02 ± 0.19 | 48 |  |
|  | WT + *exos-8* RNAi | 8.55 ± 0.34 | 48 | **<0.001^a^** |
|  | *rsks-1* + control RNAi | 7.77 ± 0.23 | 52 | **<0.001^b^** |
|  | *rsks-1* + *exos-8* RNAi | 8.32 ± 0.26 | 48 | **0.9^b^, 0.3^a^** |
| Repeat 2 | WT + control RNAi | 6.13 ± 0.2 | 47 |  |
|  | WT + *exos-8* RNAi | 9.69 ± 0.43 | 47 | **<0.001^a^** |
|  | *rsks-1* + control RNAi | 8.45 ± 0.25 | 54 | **<0.001^b^** |
|  | *rsks-1* + *exos-8* RNAi | 8.66 ± 0.31 | 56 | **0.051^b^, 1^a^** |
| Repeat 3 | WT + control RNAi | 6.55 ± 0.19 | 52 |  |
|  | WT + *exos-8* RNAi | 13.08 ± 0.39 | 56 | **<0.001^a^** |
|  | *rsks-1* + control RNAi | 10.14 ± 0.33 | 48 | **<0.001^b^** |
|  | *rsks-1* + exos-8 RNAi | 12.82 ± 0.43 | 51 | 1^b^, <0.001^a^ |
| **3E** |  |  |  |  |
| Repeat 1 | Control RNAi | 6.21 ± 0.17 | 52 |  |
|  | *exos-8* RNAi | 10.79 ± 0.33 | 56 | **<0.001^a^** |
|  | *fat-7* RNAi | 6.48 ± 0.15 | 54 |  |
|  | *exos-8 + fat-7* RNAi | 7.7 ± 0.23 | 58 | **<0.001^d^** |
|  | Control RNAi + OA | 6.62 ± 0.19 | 46 |  |
|  | *exos-8* RNAi + OA | 11.91 ± 0.32 | 58 |  |
|  | *fat-7* RNAi + OA | 7.56 ± 0.2 | 51 |  |
|  | *exos-8 + fat-7* RNAi + OA | 11.86 ± 0.4 | 51 | **<0.001^e^** |
| Repeat 2 | Control RNAi | 6.47 ± 0.12 | 49 |  |
|  | *exos-8* RNAi | 10.74 ± 0.4 | 48 | **<0.001^a^** |
|  | *fat-7* RNAi | 5.21 ± 0.21 | 52 |  |
|  | *exos-8 + fat-7* RNAi | 9.07 ± 0.39 | 50 | **0.0225^d^** |
|  | Control RNAi + OA | 6.41 ± 0.14 | 48 |  |
|  | *exos-8* RNAi + OA | 11.17 ± 0.39 | 46 |  |
|  | *fat-7* RNAi + OA | 6.46 ± 0.16 | 50 |  |
|  | *exos-8 + fat-7* RNAi + OA | 11.64 ± 0.44 | 44 | **<0.001^e^** |
| **S3I** |  |  |  |  |
| Repeat 1 | WT + control RNAi | **7.78 ± 0.16** | **106** |  |
|  | WT *+ exos-8* RNAi | **9.1 ± 0.26** | **75** | **<0.001^a^** |
|  | *nuc-1* + control RNAi | **8.06 ± 0.2** | **84** |  |
|  | *nuc-1* + *exos-8* RNAi | **9.09 ± 0.24** | **75** | **1^d^** |
| Repeat 2 | WT + control RNAi | **7.55 ± 0.42** | **40** |  |
|  | WT *+ exos-8* RNAi | **9.75 ± 0.49** | **30** | **<0.001^a^** |
|  | *nuc-1* + control RNAi | **8.21 ± 0.38** | **42** |  |
|  | *nuc-1* + *exos-8* RNAi | **9.85 ± 0.5** | **30** | **1^d^** |
| 4D |  |  |  |  |
| Repeat 1 | WT + control RNAi | 8.69 ± 0.1 | 75 |  |
|  | WT *+ exos-8* RNAi | 12.02 ± 0.21 | 89 | **<0.001^a^** |
|  | *sams-1* + control RNAi | 6.3 ± 0.12 | 83 | **<0.001^b^** |
|  | *sams-1* + *exos-8* RNAi | 6.52 ± 0.13 | 84 | **<0.001^b^**  **0.6962^a^** |
| Repeat 2 | WT + control RNAi | 8.05 ± 0.13 | 83 |  |
|  | WT *+ exos-8* RNAi | 11.77 ± 0.2 | 82 | **<0.001^a^** |
|  | *sams-1* + control RNAi | 5.78 ± 0.11 | 79 | **<0.001^b^** |
|  | *sams-1* + *exos-8* RNAi | 6.04 ± 0.16 | 89 | **<0.001^b^**  **0.5^a^** |
| Repeat 3 | WT + control RNAi | 5.71 ± 0.2 | 67 |  |
|  | WT *+ exos-8* RNAi | 8.75 ± 0.34 | 54 | **<0.001^a^** |
|  | *sams-1* + control RNAi | 4.98 ± 0.16 | 63 | **0.0105^b^** |
|  | *sams-1* + *exos-8* RNAi | 4.84 ± 0.19 | 61 | **<0.001^b^**  **1^a^** |
| 4E |  |  |  |  |
| Repeat 1 | WT + control RNAi | 6.06 ± 0.67 | 42 |  |
|  | WT *+ exos-8* RNAi | 9.83 ± 0.69 | 47 | **<0.001^a^** |
|  | *sams-1* + control RNAi | 9.71 ± 0.64 | 49 | **<0.001^b^** |
|  | *sams-1* + *exos-8* RNAi | 9.39 ± 0.65 | 49 | **0.5325^b^**  **1^a^** |
| Repeat 2 | WT + control RNAi | 6.97 ± 0.62 | 48 |  |
|  | WT *+ exos-8* RNAi | 9.72 ± 0.69 | 46 | **<0.001^a^** |
|  | *sams-1* + control RNAi | 9.26 ± 0.69 | 47 | **<0.0016^b^** |
|  | *sams-1* + *exos-8* RNAi | 8.24 ± 0.73 | 47 | **0.7298^b^**  **1^a^** |
| Repeat 3 | WT + control RNAi | 6.32 ± 0.61 | 45 |  |
|  | WT *+ exos-8* RNAi | 9.81 ± 0.72 | 42 | **<0.001^a^** |
|  | *sams-1* + control RNAi | 9.76 ± 0.71 | 48 | **<0.001^b^** |
|  | *sams-1* + *exos-8* RNAi | 9.48 ± 0.6 | 48 | **0.5452^b^**  **1^a^** |
| 4F |  |  |  |  |
| Repeat 1 | Control RNAi | 7 ± 0.18 | 43 |  |
|  | *exos-8* RNAi | 9.29 ± 0.25 | 57 | **<0.001^a^** |
|  | *pmt-2* RNAi | 4.81 ± 0.17 | 31 | **<0.001^a^** |
|  | *exos-8* + *pmt-2* RNAi | 4.08 ± 0.18 | 50 | **0.0392^f^** |
| Repeat 2 | Control RNAi | 7.36 ± 0.17 | 45 |  |
|  | *exos-8* RNAi | 9.48 ± 0.31 | 47 | **<0.001^a^** |
|  | *pmt-2* RNAi | 3.91 ± 0.2 | 46 | **<0.001^a^** |
|  | *exos-8* + *pmt-2* RNAi | 4.56 ± 0.21 | 52 | **0.1503^f^** |
| Repeat 3 | Control RNAi | 6.67 ± 0.16 | 45 |  |
|  | *exos-8* RNAi | 9.52 ± 0.3 | 45 | **<0.001^a^** |
|  | *pmt-2* RNAi | 3.78 ± 0.15 | 41 | **<0.001^a^** |
|  | *exos-8* + *pmt-2* RNAi | 3.73 ± 0.17 | 49 | **1^f^** |
| 5F |  |  |  |  |
| Repeat 1 | Control RNAi | 6.84 ± 0.24 | 50 |  |
|  | *exos-8* RNAi | 9.83 ± 0.34 | 48 | **<0.001^a^** |
|  | *eif-2Bβ RNAi* | 8.95 ± 0.32 | 53 | **<0.001^a^** |
|  | *exos-8* + *eif-2Bβ* RNAi | 10.76 ± 0.28 | 52 | **0.2943^d^**  **<0.001^g^** |
| Repeat 2 | Control RNAi | 7.4 ± 0.24 | 44 |  |
|  | *exos-8* RNAi | 9.52 ± 0.33 | 44 | **<0.001 ^a^** |
|  | *eif-2Bβ RNAi* | 11.2 ± 0.34 | 50 | **<0.001 ^a^** |
|  | *exos-8* + *eif-2Bβ* RNAi | 11.81 ± 0.37 | 49 | **<0.001^d^**  **0.474^g^** |
| Repeat 3 | Control RNAi | 6.43 ± 0.12 | 49 |  |
|  | *exos-8* RNAi | 8.28 ± 0.18 | 44 | **<0.001^a^** |
|  | *eif-2Bβ RNAi* | 7.43 ± 0.18 | 44 | **<0.001^a^** |
|  | *exos-8* + *eif-2Bβ* RNAi | 8.54 ± 0.16 | 53 | **0.8614^d^**  **<0.001^g^** |
| 5H |  |  |  |  |
| Repeat 1 | Fed Control RNAi | 5.28 ± 0.14 | 47 |  |
|  | Fed *eif-2Bβ* RNAi | 6.89 ± 0.24 | 57 | **<0.001^a^** |
|  | Fasted control RNAi | 2.67 ± 0.12 | 57 | **<0.001^b^** |
|  | Fasted *eif-2Bβ* RNAi | 7.18 ± 0.24 | 56 | **<0.001^c^** |
| Repeat 2 | Fed Control RNAi | 5.18 ± 0.17 | 51 |  |
|  | Fed *eif-2Bβ* RNAi | 7.09 ± 0.16 | 67 | **<0.001^a^** |
|  | Fasted control RNAi | 2.68 ± 0.13 | 53 | **<0.001^b^** |
|  | Fasted *eif-2Bβ* RNAi | 7.23 ± 0.22 | 49 | **<0.001^c^** |
| Repeat 3 | Fed Control RNAi | 4.89 ± 0.19 | 51 |  |
|  | Fed *eif-2Bβ* RNAi | 6.87 ± 0.24 | 58 | **<0.001^a^** |
|  | Fasted control RNAi | 2.91 ± 0.14 | 53 | **<0.001^b^** |
|  | Fasted *eif-2Bβ* RNAi | 6.71 ± 0.27 | 67 | **<0.001^c^** |
| S5C |  |  |  |  |
| Repeat 1 | WT | **27.47 ± 1.46** | **93** |  |
|  | *exos-10* | **35.31 ± 2.08** | **94** | **0.0093^b^** |
|  | *sams-1* | **45.02 ± 1.98** | **89** | **<0.001^b^** |
|  | *exos-10* + *sams-1* | **35.66 ± 1.72** | **99** | **1^d^** |
| Repeat 2 | WT | **25.83 ± 1.61** | **94** |  |
|  | *exos-10* | **34.41 ± 2.09** | **80** | **0.0025^b^** |
|  | *sams-1* | **47.86 ± 2.38** | **83** | **<0.001^b^** |
|  | *exos-10* + *sams-1* | **35.3 ± 2 .21** | **87** | **1^d^** |
| **6E** |  |  |  |  |
| Repeat 1 | WT + control RNAi | 7.68 ± 0.19 | 46 |  |
|  | WT + *exos-8* RNAi | 13.02 ± 0.31 | 52 | **<0.001^a^** |
|  | *eat-2* +control RNAi | 5.46 ± 0.11 | 65 | **<0.001^b^** |
|  | *eat-2* + *exos-8* RNAi | 10.75 ± 0.22 | 64 | **<0.001^a^** |
| Repeat 2 | WT + control RNAi | 7.37 ± 0.14 | 58 |  |
|  | WT + *exos-8* RNAi | 13.28 ± 0.23 | 62 | **<0.001^a^** |
|  | *eat-2* +control RNAi | 5.4 ± 0.11 | 58 | **<0.001^b^** |
|  | *eat-2* + *exos-8* RNAi | 10.28 ± 0.26 | 52 | **<0.001^a^** |
| Repeat 3 | WT + control RNAi | 7.31 ± 0.14 | 43 |  |
|  | WT + *exos-8* RNAi | 13.2 ± 0.25 | 52 | **<0.001^a^** |
|  | *eat-2* + control RNAi | 5.61 ± 0.12 | 57 | **<0.001^b^** |
|  | *eat-2* + *exos-8* RNAi | 10.54 ± 0.23 | 65 | **<0.001^a^** |
| S6F |  |  |  |  |
| Repeat 1 | Control RNAi | 5.73 ± 0.12 | 62 |  |
|  | *exos-8* RNAi | 8.53 ± 0.3 | 51 | **<0.001^a^** |
|  | *eif-2β* RNAi | 8.63 ± 0.2 | 65 | **<0.001^a^** |
|  | *exos-8* + *eif-2β* RNAi | 8.94 ± 0.21 | 67 | **1^d^, 0.8^h^** |
| Repeat 2 | Control RNAi | 5.88 ± 0.07 | 48 |  |
|  | *exos-8* RNAi | 8.33 ± 0.26 | 59 | **<0.001^a^** |
|  | *eif-2β* RNAi | 6.91 ± 0.22 | 47 | **<0.001**^a^ |
|  | *exos-8* + *eif-2β* RNAi | 7.69 ± 0.28 | 49 | 0.322^d^, 0.038^h^ |
| Repeat 3 | Control RNAi | 4.65 ± 0.2 | 44 |  |
|  | *exos-8* RNAi | 8.2 ± 0.47 | 43 | **<0.001^a^** |
|  | *eif-2β* RNAi | 7.28 ± 0.55 | 54 | **<0.001**^a^ |
|  | *exos-8* + *eif-2β* RNAi | 6.76 ± 0.45 | 46 | 0.1763^d^, 1^h^ |
| **S6H** |  |  |  |  |
| Repeat 1 | WT + DMSO | **8.07 ± 0.3** | **70** |  |
|  | *exos-10 +* DMSO | **10.03 ± 0.2** | **89** | **<0.001^b^** |
|  | WT *+* CHX | **9.59 ± 0.39** | **58** | **<0.001**^a^ |
|  | *exos-10 +* CHX | **10.54 ± 0.21** | **94** | **0.1675**^d^ |
| Repeat 2 | WT + DMSO | **8.15 ± 0.31** | **60** |  |
|  | *exos-10 +* DMSO | **10.05 ± 0.26** | **87** | **<0.001^b^** |
|  | WT *+* CHX | **9.68 ± 0.24** | **64** | **<0.001**^a^ |
|  | *exos-10 +* CHX | **10.75 ± 0.32** | **69** | **0.0870**^a^ |
| **S7A** |  |  |  |  |
| Repeat 1 | Control RNAi | 6.3 ± 0.12 | 60 |  |
|  | *exos-8* RNAi | 7.35 ± 0.14 | 55 | **<0.001^a^** |
| Repeat 2 | Control RNAi | 7.88 ± 0.2 | 41 |  |
|  | *exos-8* RNAi | 8.77 ± 0.23 | 34 | **0.0048^a^** |
| Repeat 3 | Control RNAi | 6.72 ± 0.17 | 60 |  |
|  | *exos-8* RNAi | 8.32 ± 0.23 | 54 | **<0.001^a^** |
| Repeat 4 | Control RNAi | 7.64 ± 0.12 | 50 |  |
|  | *exos-8* RNAi | 8.21 ± 0.12 | 60 | **0.0014^a^** |
| S7B |  |  |  |  |
| Repeat 1 | Control RNAi | 19.68 ± 1.37 | 55 |  |
|  | *exos-8* RNAi | 25.34 ± 1.4 | 55 | **0.0231^a^** |
| Repeat 2 | Control RNAi | 17.84 ± 1.2 | 62 |  |
|  | *exos-8* RNAi | 27.25 ± 1.32 | 58 | **<0.001^a^** |
| Repeat 3 | Control RNAi | 18.72 ± 1.29 | 65 |  |
|  | *exos-8* RNAi | 26.21 ± 1.35 | 64 | **0.0047^a^** |
| Repeat 4 | Control RNAi | 19.33 ± 1.27 | 57 |  |
|  | *exos-8* RNAi | 25.32± 1.46 | 57 | **0.0031^a^** |
| S7C |  |  |  |  |
| Repeat 1 | *daf-2* + control RNAi | 8.37 ± 0.17 | 46 |  |
|  | *daf-2* + *exos-8* RNAi | 12.07 ± 0.35 | 51 | **<0.001^a^** |
| Repeat 2 | *daf-2* + control RNAi | 8.25 ± 0.16 | 48 |  |
|  | *daf-2* + *exos-8* RNAi | 11.88 ± 0.29 | 52 | **<0.001^a^** |

a vs same strain

b vs same RNAi or same bacteria

c vs Fasted control RNAi or WT fasted

d vs *exos-8* RNAi or *exos-10*

e vs *exos-8* + *fat-7* RNAi

f vs *pmt-2* RNAi

g vs *eif-2Bβ* RNAi

h vs *eif-2β* RNAi
