# Supplementary material for: Inhibition of the nucleolar RNA exosome facilitates adaptation to starvation
Source: PLoS Biol. 2025 May 21;23(5):e3003190. doi: 10.1371/journal.pbio.3003190 (PMC12136472; doi:10.1371/journal.pbio.3003190)
Supplement: S2 Table — Results shown are representative of at least two independent experiments. Repeats 1 were graphed in figures. (DOCX) [file pbio.3003190.s009.docx]

**S2 Table. Lifespan data. Results shown are representative of at least two independent experiments. Repeats 1 were graphed in Figures.**

| Figures | Strain/Treatment | Mean survival time  ± SEM (days) | # Worms | p value |
| --- | --- | --- | --- | --- |
| 2J |  |  |  |  |
| Repeat 1 | Fasted control RNAi | 19.28 ± 0.51 | 70 |  |
|  | Fasted *exos-8* RNAi | 22.28 ± 0.61 | 58 | **<0.001^a^** |
| Repeat 2 | Fasted control RNAi | 18.96 ± 0.61 | 68 |  |
|  | Fasted *exos-8* RNAi | 21.77 ± 042 | 86 | **<0.001^a^** |
| Repeat 3 | Fasted control RNAi | 17.02 ± 0.57 | 81 |  |
|  | Fasted *exos-8* RNAi | 21.27 ± 0.42 | 84 | **<0.001^a^** |
| **6A** |  |  |  |  |
| Repeat 1 | Control RNAi | 17.89 ± 0.52 | 84 |  |
|  | *exos-8* RNAi | 12.38 ± 0.3 | 105 | **<0.001^a^** |
| Repeat 2 | Control RNAi | 19.06 ± 0.64 | 73 |  |
|  | *exos-8* RNAi | 12.64 ± 0.34 | 104 | **<0.001^a^** |
| Repeat 3 | Control RNAi | 19.58 ± 0.71 | 68 |  |
|  | *exos-8* RNAi | 13.43 ± 0.31 | 98 | **<0.001^a^** |
| **6B** |  |  |  |  |
| Repeat 1 | Control RNAi | 15.44 ± 0.41 | 135 |  |
|  | *exos-8* RNAi | 16.39 ± 0.47 | 149 | **0.032^a^** |
| Repeat 2 | Control RNAi | 17.62 ± 0.51 | 71 |  |
|  | *exos-8* RNAi | 19.4 ± 0.64 | 61 | **0.0117^a^** |
| Repeat 3 | Control RNAi | 20.55 ± 0.54 | 101 |  |
|  | *exos-8* RNAi | 20.28 ± 0.43 | 113 | **0.2294 ^a^** |
| Repeat 4 | Control RNAi | 19.61 ± 0.68 | 62 |  |
|  | *exos-8* RNAi | 17.27 ± 0.51 | 69 | **<0.001^a^** |
| **6C** |  |  |  |  |
| Repeat 1 | *eat-2* + control RNAi | 21.64 ± 0.47 | 96 |  |
|  | *eat-2* + *exos-8* RNAi | 20.32 ± 0.54 | 99 | **0.3576^a^** |
| Repeat 2 | *eat-2* + control RNAi | 21.00 ± 0.51 | 79 |  |
|  | *eat-2* + *exos-8* RNAi | 20.63 ± 0.54 | 114 | **0.8^a^** |
| Repeat 3 | *eat-2* + control RNAi | 21.64 ± 0.6 | 73 |  |
|  | *eat-2* + *exos-8* RNAi | 20.72 ± 0.56 | **84** | **0.3^a^** |
| **6D** |  |  |  |  |
| Repeat 1 | *eat-2* + control RNAi | 20.79 ± 0.64 | 97 |  |
|  | *eat-2* + *exos-8* RNAi | 23.97 ± 0.6 | **113** | **<0.001^a^** |
| Repeat 2 | *eat-2*+control RNAi | 19.76 ± 0.77 | 54 |  |
|  | *eat-2* + *exos-8* RNAi | 23.45 ± 1.02 | **51** | **<0.001^a^** |
| Repeat 3 | *eat-2* + control RNAi | 19.8 ± 0.51 | 77 |  |
|  | *eat-2* + *exos-8* RNAi | 22.12 ± 0.53 | **84** | **<0.001^a^** |
| S7D |  |  |  |  |
| Repeat 1 | *daf-2* + control RNAi | 29.91 ± 0.88 | 111 |  |
|  | *daf-2* + *exos-8* RNAi | 19.05 ± 0.78 | 91 | **<0.001^a^** |
| Repeat 2 | *daf-2* + control RNAi | 28.57 ± 0.89 | 95 |  |
|  | *daf-2* + *exos-8* RNAi | 17.91 ± 0.58 | 95 | **<0.001^a^** |
| S7E |  |  |  |  |
| Repeat 1 | *daf-2* + control RNAi | 34.75 ± 0.76 | 95 |  |
|  | *daf-2* + *exos-8* RNAi | 29.39 ± 1.1 | 84 | **<0.001^a^** |
| Repeat 2 | *daf-2* + control RNAi | 34.53 ± 0.8 | 87 |  |
|  | *daf-2* + *exos-8* RNAi | 27.93 ± 1.18 | 94 | **0.0013^a^** |
| Repeat 3 | *daf-2* + control RNAi | 32.39 ± 0.87 | 84 |  |
|  | *daf-2* + *exos-8* RNAi | 27.15 ± 1.15 | 78 | **0.0298^a^** |

a vs same strain
