# Supplementary material for: Inhibition of the nucleolar RNA exosome facilitates adaptation to starvation
Source: PLoS Biol. 2025 May 21;23(5):e3003190. doi: 10.1371/journal.pbio.3003190 (PMC12136472; doi:10.1371/journal.pbio.3003190)
Supplement: S3 Table — (DOCX) [file pbio.3003190.s010.docx]

**S3 Table. The knockdown efficacy of RNAi.**

|  | mRNA levels | |  |
| --- | --- | --- | --- |
| Gene names | Control RNAi | RNAi treatment groups | p value |
| *exos-8* | 1 | 0.16 ± 0.02 | < 0.001 |
| *exos-4.2* | 1 | 0.14 ± 0.06 | < 0.001 |
| *eif2β* | 1 | 0.2 ± 0.08 | < 0.01 |
| *eif2Bβ* | 1 | 0.2 ± 0.04 | < 0.01 |
| *pmt-2* | 1 | 0.48 ± 0.1 | < 0.05 |
| *mtr-4* | 1 | 0.42 ± 0.11 | < 0.05 |
| *fib-1* | 1 | 0.36 ± 0.15 | 0.053 |
| *nol-56* | 1 | 0.48 ± 0.08 | < 0.05 |
| *T22H9.1* | 1 | 0.4 ± 0.12 | < 0.05 |
| *sams-1* | 1 | 0.03 ± 0.02 | < 0.001 |
| *let-363* | 1 | 0.35 ± 0.02 | < 0.001 |
