# Supplementary material for: Inhibition of the nucleolar RNA exosome facilitates adaptation to starvation
Source: PLoS Biol. 2025 May 21;23(5):e3003190. doi: 10.1371/journal.pbio.3003190 (PMC12136472; doi:10.1371/journal.pbio.3003190)
Supplement: S4 Table — (DOCX) [file pbio.3003190.s011.docx]

**S4 Table. Primers for cRT-PCR and qPCR.**

| **Primers** | **Sequences** |
| --- | --- |
| 18S RT (cRT-PCR) | GTATAGTTGCATGTATTAGCTCCAG |
| 18S F (cRT-PCR) | GGTATTGTAATTATTGCCCTTAAACG |
| 18S R (cRT-PCR) | TCAGGTATTACGCAGACATATAG |
| *fat-7* Forward | CCGATAATCTCATCACAA |
| *fat-7 Reverse* | ATTCTCATTGGTGTGGTT |
| *sams-1* Forward | CGGATATGCAACCGACGA |
| *sams-1* Reverse | GACCACAACAGTGTGAACG |
| *mel-32* Forward | CACCAACAACGAGAACATT |
| *mel-32* Reverse | GCATCCATAACAGCCTTG |
| *mthf-1* Forward | TCATCAACTACATTACTCAAGC |
| *mthf-1* Reverse | AATTCCATTCTCATTACACCAA |
| *metr-1* Forward | TTACGATACGGCGATTGA |
| *metr-1* Reverse | GAGGATATGAGACGAAGGAA |
| *mtrr-1* Forward | ATGAAGGAAGAGGAAGAGTTAT |
| *mtrr-1* Reverse | ACGAGACGATGAAAGGTT |
| *ahcy-1* Forward | ACAGGTTGACCGTTACAC |
| *ahcy-1* Reverse | ACATCCAAGGTTGACAAGA |
| *pmt-2* Forward | ATAAGGTGACCGAGGGAC |
| *pmt-2* Reverse | TCGGCGTTGCGAATAGT |
| *snb-1 Forward* | GCAAGTATTGGTGGAAGA |
| *snb-1 Reverse* | ACGATGATGATAATAAGAATGAC |
| *hsp-4* Forward | AGGAGCAAAAGAAGGAACTTGA |
| *hsp-4* Reverse | GCAGCAAGTAGTTGAAGGCA |
| *hsp-6* Forward | GGACGCTGGAGATAAGATCATCG |
| *hsp-6* Reverse | TGGACTTGACCTCGAAGACG |
| *hsp-16.2* Forward | TCCATCTGAGTCTTCTGAGATTGTT |
| *hsp-16.2* Reverse | TGATAGCGTACGACCATCCAAA |
| *mtl-1* Forward | GGCTTGCAAGTGTGACTGC |
| *mtl-1* Reverse | TTTCTCACTGGCCTCCTCAC |
| *gst-4* Forward | CTCTTGCTGAGCCAATCCGT |
| *gst-4* Reverse | TGGCCAAATGGAGTCGTTGG |
| *exos-8* Forward | TTTGGATGCAGCTGTGTGCG |
| *exos-8* Reverse | CAATTGTCGATTCGTCGCCC |
| *exos-4.2* Forward | AGTTTTGGCACAAATCACAGGA |
| *exos-4.2* Reverse | ATACTGCAGACGCCAGTTGA |
| *let-363*Forward | CGAGCGGCATCGTAAACAAG |
| *let-363* Reverse | GGCTCGTCTTTCAGCTCACT |
| *eif-2beta* Forward | ATGGCTGACGATTTGGGTCT |
| *eif-2beta* Reverse | TCGCCCAAGCCATCTTCTAC |
| *eif-2Bbeta* Forward | ATGCCTGAAGCCTCGAAAGT |
| *eif-2Bbeta* Reverse | GCGAGCACGTAGACTGGAAC |
| *nol-56* Forward | CCATCTCTGAAGGTCTCGCC |
| *nol-56* Reverse | TTCAGTAAGCGAACCCGCAA |
| *T22H9.1* Forward | GTTCACCGAGGAGGGCAAATA |
| *T22H9.1* Reverse | CTCCTTGTGAATTGTTGCCATT |
| *fib-1* Forward | GGAGTTGCCAAGAAGAGACCA |
| *fib-1* Reverse | GACATCGACCATTCCGACAA |
| *mtr-4* Forward | ATGCGAGATAAAGAGCGCGG |
| *mtr-4* Reverse | GGTTGTTGCTTGATGCTGGC |
