# Supplementary material for: Inhibition of the nucleolar RNA exosome facilitates adaptation to starvation
Source: PLoS Biol. 2025 May 21;23(5):e3003190. doi: 10.1371/journal.pbio.3003190 (PMC12136472; doi:10.1371/journal.pbio.3003190)
Supplement: Attachment — Submitted filename: Point by point response.docx [file pbio.3003190.s015.docx]

**We sincerely thank the reviewers for their constructive comments, which have significantly enhanced the quality of our manuscript. The key issues addressed include: (1) validating several major findings in the manuscript using RNA exosome mutants; (2) characterizing RNA exosome suppression under nutritional deficiency by establishing an assay to examine rRNA processing, a process mediated by the RNA exosome; (3) demonstrating that nucleolus size is not involved in the effects of RNA exosome inhibition; and (4) addressing additional concerns through supplementary experiments and textual revisions.**

*Rev. 1:*

*In this article, Feng at all investigate the role of nucleolar RNA exosome in stress response and lifespan in C. elegans. The authors describe the depletion of RNA exosome subunits and hence inactivation of the organelle in response to various stressors such as starvation and oxidative stress. they also demonstrate that RNAi depletion of two of these subunits increases survival upon exposure to these stressors. RNAi of one of these subunits (exos-8) throughout development and adulthood shortens lifespan whereas postdevelopmental inhibition does not. They explore this phenomenon by conducting RNAseq and subsequently examine the impact of exosome depletion treatments on fat levels, 1 Carbon chain-pathway genes, and based on these experiments propose that exosome inhibition 'rewires' 1C metabolism to reduce translation and increase lipogenesis. While the study is based on some really interesting observations on the role of RNA exosomes in stress response, there are several significant shortcomings that arrest enthusiasm for it.*

*1. The links between exos- gene RNAi and lipid metabolism, translation, mTOR and subsequently stress response are derived from (a) cherry picked observations and (b) inadequate experimental approaches, to make broad conclusions that are not all merited.*

*a. eg., if top RNAseq classes were used to investigate the underlying metabolism, then the case for 'proteolysis/proteosomal function' and 'transmembrane signaling' are much more stronger pathways than lipid metabolism. lipid metabolism is the 'mechanistic' pathway.*

**Response:** We apologize for not making our rationale clear in the initial manuscript. We selected metabolism for mechanistic study because: (1) metabolic genes and metabolites are among the most influential factors known to regulate stress responses in C. elegans; (2) as shown in Figure 2, the RNA exosome responds to nutrient stress to modulate stress resistance. We thus speculated that metabolic changes might act downstream of the RNA exosome to coordinate metabolic adaptation and stress resistance. These two points underlie our focus on metabolic changes, and we have revised the paragraph (Page 8, line 221-226) to clarify this reasoning.

*b. A major drawback is that the study relies almost exclusively on RNAi and no mutant analysis (or other forms of gene inactivation such as Auxin-mediated degradation) and in some cases double RNAi which is even less reliable. Similarly, using fat staining/levels alone to draw conclusions about lipogenesis being triggered by RNA exosomone inactivation. Alterations in overall fat levels could be due to other reasons besides increased lipogenesis. For eg., knockdown of nhr-49, which mediates the lipolytic process of b-oxidation, also causes reduced ORO staining/fat levels.*

**Response:** We thank the reviewer for these valuable suggestions. In response to your comments regarding the lack of mutant analysis, we have included additional experiments using exos-10 mutants, which target the catalytic subunit of the RNA exosome. These experiments confirmed the main findings of our study by showing that *exos-10* mutants (1) promoted stress resistance (new Fig. S1H-S1K), (2) increased fat accumulation (new Fig. S4C), (3) enhanced fat accumulation and stress resistance during starvation (new Figs. S4H and S2J), (4) regulated fat accumulation and stress resistance via the one-carbon metabolism gene sams-1 (new Figs. S5B-S5C), (5) reduced translation levels (new Fig. S6D), and (6) regulated stress resistance through translational repression (new Fig. S6G). We observed that exos-10 mutants behaved similarly to RNAi knockdown of structural subunits (*exos-4.2* and *exos-8*), thereby corroborating our conclusions.

Regarding the interpretation of fat accumulation results, we agree with the reviewer that an increase in fat alone does not imply increased lipogenesis. The correct interpretation is that RNA exosome inhibition led to a remodeling of lipid metabolism, including the upregulation of genes involved in both lipid synthesis and catabolism, along with an increase in fat levels. Specifically, upregulation of fat-6, a lipid synthesis gene, contributed to enhanced stress resistance. We have revised the relevant conclusions (Page 9, line 254-256) to more accurately reflect these findings.

*2. There are many new studies that have demonstrated that nucleolus size itself impacts, and is reflective of, lifespan and stress response. This is bound to have strong interactions with/impact on RNA exosome in the nucleolus and is not considered at all.*

**Response:** We thank the reviewer for the valuable suggestion. Given that the RNA exosome is primarily located in the nucleolus, we agree that it may influence stress responses through nucleolus size. To test this hypothesis, we used the nucleolus marker FIB-1::mCHERRY and found that RNA exosome repression did not affect nucleolus size (new Fig S3H). Additionally, since a smaller nucleolus is typically associated with enhanced stress resistance and increased longevity, we examined ncl-1 mutants, which have an enlarged nucleolus, and observed that RNA exosome suppression still led to increased stress resistance (new Fig S3I) in *ncl-1* mutants. These findings suggest that exosome repression does not regulate the stress response via changes in nucleolus size.

*3. In some cases, key controls are missing that make interpretations difficult. Eg., if starvation (and other stressors) depletes nucleolar exosome, then the fed/un-stressed controls are critical (eg. Fig. 1B-E) to understand how molecular depletion of exos subunits (as compared to starvation driven one) are similar or different. Most stressors cause reduced survival which means that simply exosomal depletion doesn't cause increased resistance. Such key controls are missing in many panels.*

**Response:** This is an important concern. Regarding starvation, Figures 2I and S2J compares the effect of RNA exosome repression on stress resistance under fed versus starved conditions. The data show that RNA exosome repression effectively prevents the inhibitory effect of starvation on stress resistance, suggesting that RNA exosome deletion plays a key role in maintaining stress resistance during starvation. Although starvation itself inhibits the RNA exosome, this does not contradict its inhibitory effect on stress resistance. As noted in the introduction, starvation induces multiple stressors, such as oxidative stress, which contribute to reduced stress resistance. RNA exosome inhibition serves as a protective response against such stressors.

The reviewer also raised a valid point regarding unstressed controls. First, since the RNA exosome is depleted by stressors, it is not feasible to establish an unstressed control to test the effect of RNA exosome suppression (via RNAi or mutation) on stress resistance, as this inherently requires the presence of stressors. Second, we have examined the effect of RNA exosome inhibition on the survival of animals under unstressed conditions through lifespan experiments (Figure 6). These experiments revealed that RNA exosome inhibition reduced the lifespan of wild-type animals. This suggests that RNA exosome inactivation specifically enhances stress resistance but does not generally improve survival under unstressed conditions. Notably, while stressors deplete the RNA exosome, this does not contradict the reduced survival caused by these stressors. Instead, RNA exosome inhibition serves as an adaptive mechanism to help the organism manage stress. Consistently, RNA exosome inhibition improved survival under stress conditions (Figures 1B–1E, and new S1H-S1K).

*4. Using ORO or Nile Red-derived fat levels is not sufficient to conclude that lipid biosynthesis is upregulated, especially since expression of both lipogenic and lipolytic genes were elevated.*

**Response:** Please see our response to your comment 1b.

*Rev. 2:*

*The study elucidates how cells adapt to nutrient scarcity by modulating the nucleolar RNA exosome, a crucial RNA surveillance component. It shows that inactivating the RNA exosome enhances stress resistance, improves fitness under nutrient scarcity, and promotes longevity in animals under dietary restriction by reallocating energy resources. This adaptation involves a shift in cellular metabolism, favoring lipid biosynthesis and energy conservation through reduced protein synthesis, without directly regulating stress response genes. Several issues need to be resolved prior to publication.*

*1. It is crucial to clarify whether the observed enhancement in stress resistance is dependent on the reduced endonuclease or exonuclease activity within the RNA exosome complex. This specificity is essential for understanding the mechanistic basis of the proposed phenomenon.*

**Response:** We thank the reviewer for raising this important concern. Based on previous and new data, we propose that the loss of exonuclease activity of the RNA exosome is sufficient to trigger stress resistance. In other organisms, the DIS-3 catalytic subunit (RRP44) exhibits both endonuclease and exonuclease activities, while the EXOS-10 subunit (EXOSC10) is limited to exonuclease function. The *C. elegans* EXOS-10 subunit shares conserved functional domains with its orthologs, suggesting a conserved exonuclease function. We confirmed the exonuclease activity of EXOS-10 in the nucleolus in processing rRNA, as *exos-10* mutants accumulate rRNA intermediates (new Figs. 2E-2F). Similar to RNAi knockdown of core exosome subunits, *exos-10* mutants displayed enhanced stress resistance, lipid accumulation, and translational suppression (new Figs. S1H-S1K, S4C, and S6D), further supporting the critical role of RNA exosome exonuclease activity in these processes.

At present, it remains unclear whether C. elegans DIS-3 possesses both endonuclease and exonuclease activities. A comprehensive analysis of the DIS-3 subunit is essential to address this question, which can provide valuable insights into whether endonuclease activity contributes to the RNA exosome’s regulation of stress resistance. We incorporate these interpretations into the revised discussion section (Page 14-15, line 408-421)

*2. Figure S1 does not specify the developmental stage of the worms at the time the fluorescent images were captured. Normally, the control group under standard conditions (untreated and prior to worm aging) should not display significant fluorescence. Nonetheless, the fluorescence intensity is unexpectedly high in the control samples for hsp-6p::GFP and T24B8.5p::GFP shown in Figure S1. To gain a more accurate insight into the cellular response following RNA exosome component knockdown, I suggest employing qPCR analyses to directly measure the expression levels of endogenous genes for the endoplasmic reticulum, mitochondrial, and oxidative and heat stress responses.*

**Response:** Thank you for your valuable suggestions. Our fluorescence images were taken from day 1 adults, and we have added this detail to the revised method. For hsp-6p::gfp and hsp-16.2p::gfp, as you noted, background fluorescence was initially very weak, which obscured the enhancement effects of exosome RNAi. To address this, we applied a stressor (Antimycin for hsp-6p::gfp and 35°C for hsp-16.2p::gfp) to increase fluorescence intensity and observed that exosome RNAi did indeed enhance their expression. We apologize for omitting this important information in the original manuscript. Additionally, we have removed T24B8.5p::GFP data from the revised manuscript, as we did not assess pathogen stress resistance relevant to this reporter.

Following your suggestion, we also examined the effect of exosome RNAi on the expression of these endogenous stress genes under non-stress conditions using qPCR. The results indicated that exosome RNAi increased the expression of these genes (new Figure S1G), with the exception of the ER stress gene hsp-4, which was not significantly upregulated, though the reason for this remains unclear. These findings support our conclusion that RNA exosome repression activates stress responses.

*3. The choice of using exos-8 and exos-4.2 RNAi for experiments related to stress resistance, fat accumulation, and subsequent RNA sequencing to represent "RNA exosome inactivation" raises concerns. Why were mutants of these genes not utilized instead? Furthermore, considering that the actual catalytic components of the RNA exosome are dis-3 and crn-3, it is unclear why the experiments focused on the structural subunits exos-8 and exos-4.2 instead of these core catalytic subunits. Inactivation or knockdown of the catalytic subunits might more accurately reflect the effects of RNA exosome inactivation and could provide clearer insights into the mechanism and phenotype alterations observed.*

**Response:** We thank the reviewer for these valuable suggestions. In response to your comments, we have conducted additional experiments using exos-10/crn-3 mutants, which target the exosome's catalytic subunit. We were unable to identify a dis-3 mutant with normal development and reproduction. The new experiments confirmed the main findings of our study, showing that *exos-10* mutants (1) promoted stress resistance (new Fig. S1H-S1K), (2) increased fat accumulation (new Fig. S4C), (3) enhanced fat accumulation and stress resistance during starvation (new Figs. S2J and S4H), (4) regulated fat accumulation and stress resistance via the one-carbon metabolism gene sams-1 (new Figs. S5B-S5C), (5) reduced translation levels (new Fig. S6D), and (6) regulated stress resistance through translational repression (new Fig. S6G).

*4. The characterization of "RNA exosome inactivation" in Figures 2 and Figure S2, referencing a 2021 Nucleic Acids Research paper, equates this inactivation with the nuclear localization change of "GFP::EXOS-1." However, the referenced study indicates that this localization change impacts only a subset of RNA exosome functions, specifically risiRNA production, and does not denote the complete inhibition of RNA exosome activity. Therefore, equating "RNA exosome inactivation" solely with "GFP::EXOS-1" nuclear localization changes is misleading. A more accurate characterization of RNA exosome inactivation should involve the inactivation of its core catalytic subunits (dis-3 & crn-3), not merely the localization changes of "GFP::EXOS-1."*

**Response:** This is a very important concern. To address it, we first analyzed the subcellular localization of EXOS-10, the catalytic subunit of the RNA exosome, in response to starvation. Using an endogenous GFP-tagged EXOS-10, we showed that EXOS-10 was depleted from the nucleolus upon starvation (new Fig. 2C), akin to the other structural subunits.

Then, we studied the activity of the RNA exosome by examining rRNA processing, a central function of the RNA exosome in mammals. Using circular RT-PCR, a method to identify and measure rRNA intermediates, we detected two 18S pre-rRNA and found that mutation of *exos-10* caused accumulation of them (new Figs. 2E and 2F), validating the function of *C. elegans* RNA exosome in rRNA processing. We then examined rRNA processing during starvation and hypothesized that if starvation suppresses RNA exosome activity, it would lead to the accumulation of the same pre-rRNAs observed in exos-10 mutants. Consistent with this hypothesis, starved animals showed significant accumulation of these 18S pre-rRNAs (new Fig. 2G). Collectively, these findings suggest that the RNA exosome is repressed by starvation, likely via their nucleolar depletion.

*5. The manuscript lacks data on knockdown efficiency for the RNAi experiments on C. elegans genes, which is essential for validating the results.*

**Response:** We have validated the RNAi efficiency and included these data in Table S3 of the revised manuscript.

*6. The manuscript does not specify the starvation treatment details in the methods or main text. Since the starvation protocol can significantly affect the experimental outcomes, it is crucial to elucidate this to ensure the study's validity. Please provide these details.*

**Response:** We apologize for omitting this important information in the initial manuscript and have now included it in the figure legends.
