# Supplementary material for: Inhibition of the nucleolar RNA exosome facilitates adaptation to starvation
Source: PLoS Biol. 2025 May 21;23(5):e3003190. doi: 10.1371/journal.pbio.3003190 (PMC12136472; doi:10.1371/journal.pbio.3003190)
Supplement: Attachment — Submitted filename: Response to reviewers.docx [file pbio.3003190.s017.docx]

*Rev. 1:*

*In the revised version of their article, Feng et al have addressed my concerns significantly though not completely. I am happy to support publication of the manuscript in PLoS Biology. I have a few comments/suggestions below that I recommend be applied to the article before publication.*

**Response:** We thank the reviewer for his/her positive comment on the revisions to our manuscript.

*1. It will help if the figure legends of the survival graphs provide more information in terms of mean survival value, SEM, P values. It is great that the Suppl. Tables list these data from the different trials, but adding the specific values in the legend (or indicating which trial # is being reported in the figure) will improve the reading experience.*

**Response:** According to your suggestion, we have indicated in both the figure legends and table legends that survival data from Replicate 1 were used for plotting in the figures.

*2. Similarly, for experiments such as ORO staining and qPCRs, it is important to indicate how many trials/biological replicates were performed and what the no. of animals in each was. And, if the quantification is derived from combining all or select replicates.*

**Response:** We appreciate your suggestions. In revising the manuscript, we have incorporated the number of experimental replicates and the data sources used for quantification in the Methods section.

*3. I strongly recommend moving the exos-10 mutant data (Fig. S1) to the main manuscript along with the schematic of the exosome.*

**Response:** We agree with your suggestion and have moved these data to Figure 1.

*4. It is not clear how Fig. 3A, B is/are different from Fig. 3C, D?*

**Response:** We apologize for the lack of clarity in the original description. Figure 3A/3B present broad functional categorization (Category One) of all differentially expressed genes using Wormcat, where metabolic processes are grouped as a single class. Figure 3C/3D demonstrate refined subcategorization (Category Two), in which metabolic pathways are further divided into multiple specialized subtypes, with only the most significantly enriched metabolic subclasses being displayed. We have revised the figure legends to enhance clarity regarding this hierarchical classification.

*5. Where are these exosome genes expressed? Were the reporter constructs used in Fig. 1 etc,. driven under endogenous promoters or in specific tissues? Which tissues was the imaging done on- one or more? This information is either not included or poorly accessible. Please include this. It will help if there is also some discussion about the spatial aspects of this regulatory phenomenon.*

**Response:** The RNA exosome gene is expressed in multiple tissues of *C. elegans*. In our study, its expression in intestinal cells was shown, as these cells serve as the primary sites for lipid synthesis/storage and the main tissue for stress-responsive gene expression in *C. elegans*. All fluorescent reporters were driven by endogenous promoters. Nuclear fluorescence images were captured from intestinal cells, whereas stress reporter genes and lipid staining were analyzed through whole-animal imaging, showing that stress-responsive gene expression and lipid accumulation predominantly occur in intestinal tissue.

We have incorporated these technical details into the figure legends to ensure readers can accurately interpret the experimental information. Additionally, following the reviewer's suggestion, the revised manuscript discusses the expression patterns of the RNA exosome. It specifically highlights the need for future investigations to address the following questions: Through which tissue hub does the RNA exosome coordinate organism-wide metabolic and stress responses? Do its regulatory mechanisms involve cell-non-autonomous mechanisms?
